# Resilience and Extrinsic Motivation as Mediators in the Relationship between Fear of Failure and Burnout

**DOI:** 10.3390/ijerph20105895

**Published:** 2023-05-20

**Authors:** Murat Yıldırım, Ömer Kaynar, Francesco Chirico, Nicola Magnavita

**Affiliations:** 1 Department of Psychology, Faculty of Science and Letters, Agri Ibrahim Cecen University, 04100 Ağrı, Turkey; 2 Sports Science Faculty, Muş Alparslan University, 49250 Muş, Turkey; 3 Post-Graduate School of Occupational Health, Università Cattolica del Sacro Cuore, 20123 Rome, Italy; 4 Health Service Department, Italian State Police, Ministry of the Interior, 00198 Milan, Italy

**Keywords:** fear of failure, resilience, extrinsic motivation, burnout, Turkish athletes

## Abstract

Athletes with fear of failure are at risk of developing the symptoms of a wide range of psychological problems, including burnout. Understanding the risks and protective factors of athletes’ psychological health is an essential step in tailoring strategies and interventions to promote athletes’ psychological and mental health. This study examined the mediating roles of resilience and extrinsic motivation in the relationship between fear of failure and burnout among Turkish athletes. The study included 335 young athletes (93.4% males) whose ages ranged from 18 to 55 years (*M* = 24.95, *SD* = 8.22). Participants completed the self-reported measures of fear of failure, resilience, extrinsic motivation, and burnout. The analysis revealed that fear of failure had significant predictive effects on resilience, extrinsic motivation, and burnout. Resilience and extrinsic motivation also had significant predictive effects on burnout. The mediation analysis results showed that both resilience and extrinsic motivation partially mediated the effect of fear of failure on athlete burnout. The findings of the study provide a better insight into the underlying mechanisms between fear of failure and athlete burnout by considering resilience and extrinsic motivation as mediators. These results suggest that the adverse impact of fear of failure on athlete burnout can be mitigated by cultivating resilience and hindering extrinsic motivation.

## 1. Introduction

Fear of failure can be a risk factor diminishing the psychological health of athletes. In particular, extreme fear of failure is a major barrier to success for athletes who endeavour to achieve the highest level of excellence. The concept of fear of failure refers to the tendency to appraise threats in situations where the probability of failure is present [1]. According to researchers and achievement motivation theorists, fear of failure is learned at early ages between 5 and 9 years through practices of socialisation [2]. Individuals with high levels of fear of failure tend to exhibit destructive self-talk such as self-blame [3].

Fear of failure is a common self-destructive feeling, and most athletes have difficulties overcoming these negative experiences. Athletes experience fear of failure at different times and in various contexts, especially when they are concerned about not achieving what they wish and have intensively worked to acquire, such as winning a wrestling championship [1]. Research findings have shown that fear of failure is related to various factors such as a maladaptive aspects of perfectionism [4] and personality characteristics such as avoidance temperament [5]. Fear of failure is also associated with a wide range of social and individual factors, such as age, goal attainment, types of sport, perceived competence, self-control, self-compassion, shame coping, well-being, interpersonal behaviour, and sports performance [6,7,8]. Furthermore, a greater experience of fear of failure is linked with ineffective coping strategies to deal with failure experiences [9]. As a result of this, athletes who do not have effective coping abilities to overcome such situations tend to experience negative effects and poor performance, and even may subsequently drop out of the sport [10]. As such, athletes need to be equipped with and use a wide range of character strengths and coping strategies to cope with these demands. The inability to deal with these situations may result in serious problems in athletes’ performance.

Taylor et al. [1] highlighted that examination of the fear of failure in sports is necessary to advance knowledge and gain a comprehensive understanding of how best to design interventions focused on diminishing the fear of failure within individuals and better understanding the reciprocal effects within their social network. Therefore, examining the impact of fear of failure on psychological health and the possible mechanisms between this would be useful for research and practice. As such, the underlying mechanism between fear of failure and burnout with resilience and motivation as possible mediators were examined in this study.

In the wider literature, the concept of burnout has been conceptualised in terms of three main dimensions that include emotional exhaustion (loss of energy, wearing out, debilitation, fatigue, and depletion), reduced personal accomplishment (diminished productivity, an inability to deal with stress, and lower morale), and depersonalisation (loss of idealism, withdrawal, and irritability) [11,12]. In the context of sports, burnout refers to a syndrome characterised by emotional and physical exhaustion, a decreased sense of accomplishment or success, and sport devaluation [13]. This multifaceted framework of burnout suggests the importance of individual differences in the experience of stress in various contexts [12], including sports, by involving one’s perception of the self and others [13].

Athletes put a lot of physical and psychological effort into attaining success and maintaining their success for a long period of time in sports environments requiring intense competition [14]. In the face of stressful situations such as sports competitions, a positive state of psychological health of athletes serves a critical role in terms of producing better sportive performance. In such stressful situations, athletes may suffer from sport-related burnout that involves an inability to respond to stress corresponding to physical exercises and competition [13]. As such, burnout should be taken into account as a major concept as its adverse impact on well-being and mental health outcomes cannot be neglected [15,16], like many other psychosocial factors [17,18,19,20].

Researchers have highlighted that it is critical to prevent and mitigate athlete burnout [21]. A systematic review paper including 58 published scientific studies reported a wide range of demographic, psychological, and situational factors affecting athletes’ burnout either positively or negatively. These factors include motivation, perceived control, coping, resilience, social support, enjoyment, anxiety, perceived stress, mood disturbance, parental influence, coach behaviour, identity, and training load [22]. Another recent systematic review study demonstrated that various factors could lead to athlete burnout, such as perfectionistic concerns, chronic stress, and reduced self-determined motivation [21]. A high level of fear of failure was found to be associated with high levels of burnout and psychological distress in athletes competing in high-level sports [21]. Furthermore, resilience has been linked to reduced burnout [23,24]. Therefore, psychological resources such as resilience can be used to mitigate the perfectionistic mindset as effective coping strategies in the prevention and treatment of athlete burnout [21].

Resilience is an important psychological factor that is assumed to mitigate the impact of fear of failure on burnout. The concept of resilience is defined as a dynamic process that concerns how people respond to stressors, threats, risks, and challenges [25,26]. Researchers conceptualise resilience in different ways, stressing its protective and mitigating roles in that resilience minimises the adverse effects of events, improves adjustment and the adaptation of an individual to the environment, enhances the ability for problem-solving, and contributes to positive personality traits, well-being, and mental health [27].

We have assumed that resilience is an effective mitigating factor to reduce burnout in athletes. As such, we considered a socio-ecological perspective to better understand the fundamental process of resilience as a potential mediator in the relationship between fear of failure and burnout. According to Ungar [28], resilience, based on the social ecology approach, is an individual’s capacity to seek resources and cope with challenges in a culturally meaningful way to maintain and protect well-being in the face of any adversities, challenges, or events. In his social ecology theory of resilience, Ungar [28] highlighted the importance of individual capacities and resources to adopt resilient pathways. For example, resilience is related to the well-being and psychological health of people against stressors or challenges [29,30].

Given that resilience has a mitigating and protective role in stressful situations [29], it is important to incorporate these aspects when investigating the essential factors of well-being, motivation, and burnout, which differ based on diverse populations, contexts, and cultures. The existing literature showed that both internal and external factors significantly contribute to the ability to have better well-being and psychological health [29,31,32]. Internal factors are linked with the personal characteristics of a person, having a meaningful effect on a person’s psychological health and ability to cope with stressful situations, such as cognitive skills, self-worth, self-regulation, positive attitude toward the self, motivation, and personal values [33,34]. External factors, on the other hand, influence the ability of a person to be resilient while dealing with stressful situations or adversity. For instance, a strong social support system promotes and reinforces coping skills among people to adopt resilient pathways [35]. In the context of athletes, resilience can enhance the ability to recover from hardship and stressors both at the personal level and the sport-environmental level [36]. Therefore, resilience can be trained, developed, and fostered in athletes.

The concept of motivation can be best understood within the Self-determination Theory (SDT) [37,38], which asserts that every individual has the ability to make choices and manage their own life. In this theory, such an ability is vital in well-being and psychological health through enabling individuals to believe that they have explicit control over their choices and lives. Self-determination has a positive impact on motivation, by which individuals feel more motivated to engage in activities and develop toward universal psychological needs which can be achieved through the experience of autonomy, competence, and relatedness. In the tradition of SDT, motivation is differentiated into intrinsic and extrinsic motivation [37,38]. Intrinsic motivation refers to individuals’ tendencies to perform an activity which is intrinsically interesting and spontaneously satisfying, while extrinsic motivation refers to engagement in an activity because it causes some separate outcomes [39]. Those who are intrinsically motivated participate in activities just because of the experience of positive feelings, pleasures, and enjoyment derived from the activities themselves. To master optimal challenges, those people willingly perform what they are doing with high curiosity and novelty. On the other hand, those who are extrinsically motivated tend to perform behaviours or get involved in activities which lead to tangible rewards and distance from pain or punishment [39].

In the context of sports, motivation is studied to understand physical activities and performance. In this regard, a substantial amount of work has been carried out on motivation in order to comprehend why some athletes demonstrate a long-lasting tendency and desire to pursue their sports activities, while others quit or lose interest [40]. Studies have shown that athletes can be motivated by various external factors including evaluations, rewards, and pressure from friends, parents, and coaches [41]. Furthermore, motivation is also found to be related to long-time commitment and the risk of suffering from bulimic symptoms [42], sport satisfaction, subjective vitality, and an absence of negative physical symptoms [43]. A lack of motivation or solid achievement goals is positively linked to fear of failure, which has the potential to lead to a wide range of maladaptive self-protective strategies [44]. Moreover, a greater autonomous form of motivation can be achieved through the fulfilment of psychological needs, and such a fulfilment leads to greater personal and sports outcomes [40].

**Figure 1:**
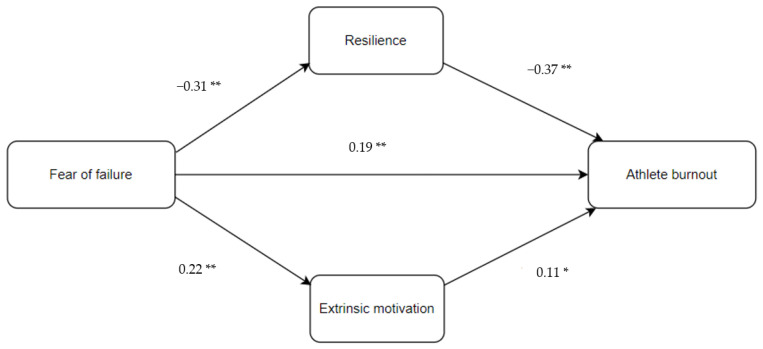
Structural model showing the associations between the variables (** *p* < 0.001, * *p* < 0.05).

Previous research has emphasised that a comprehensive understanding of fear of failure in athletes is a critical step for promoting their psychological well-being, quality of engagement, motivation, sports performance, and social development [45]. Studies have typically been conducted to examine the direct impact of fear of failure on mental health outcomes (e.g., burnout) in sports, with a lack of attention given to the investigation of possible mechanisms that may explain this association [46]. In this regard, two important mechanisms, namely, resilience and motivation, may be of importance. From an applied point of view, it is important to obtain a better understanding of psychological factors (e.g., resilience and motivation) in explaining the association between fear of failure and burnout of athletes. This has a significant potential for developing models of psychological care and management in this population, which may in turn promote positive sports-related outcomes. Such a model and an understanding are necessary to inform sports authorities and provide guidance and support for the sport’s practitioners, such as sports psychologists, coaches, and medical staff, in designing coping strategies for athletes and, in turn, improving their psychological health, and reducing burnout. On the basis of our literature review sketched above, a structural model was proposed, as shown in Figure 1. Therefore, this study aims to examine mediating roles of resilience and motivation in the relationships between fear of failure and burnout among athletes. In light of this structural model, we proposed three main hypotheses:

**H1.**  *Fear of failure would have a direct negative impact on resilience and a direct positive impact on extrinsic motivation and burnout.*

**H2.**  *Resilience would have a direct negative impact on burnout, while extrinsic motivation would have a direct positive impact on burnout.*

**H3.**  *Resilience and extrinsic motivation would mediate the association between fear of failure and burnout.*

## 2. Method

### 2.1. Participants

The requisite sample size was based on the rule of thumb that a sample size between 115 and 285 is sufficient to detect an indirect effect among the variables [47]. The sample for this study included 335 young Turkish athletes (93.4% males) whose ages ranged between 18 and 55 years, with a mean age of 24.95 years (*SD* = 8.22). Most athletes had a bachelor’s degree (52.54%), were single (72.24%), national championship (37.01%), national athletes (50.15%), and had been doing sports actively for 10 years and above (46.57%). Detailed demographic information is presented in Table 1. Athletes aged 18 years and above who were engaged in active sports were included in the study. Those who did not meet these criteria were not allowed to take part in the study.

### 2.2. Measures

#### 2.2.1. Fear of Failure

The Performance Failure Appraisal Inventory (PFAI) [48] is a multidimensional instrument of threat appraisals relate to fear of failure. Participants were required to answer how strongly they believed each of the aversive consequences of failure was likely to happen to them following failure. The PFAI included 25 self-reported items grouped into five domains: (i) fear of experiencing shame and embarrassment (seven items), (ii) fear of having an uncertain future (four items), (iii) fear of devaluing one’s self-estimate (four items), (iv) fear of important others losing interest (five items), and (v) fear of upsetting important others (five items). Only an experiencing shame and embarrassment subscale was used for the purpose of this study. Each item was rated on a 5-point Likert scale from 1 (do not believe at all) to 5 (truly believe). A composite fear of failure score could be obtained by summing the individual items on each sub-scale. Turkish validation of the PFAI produced good reliability and validity evidence [49]. In this study, Cronbach’s alpha coefficient for the PFAI was 0.82.

#### 2.2.2. Burnout

The athlete Burnout Questionnaire (ABQ) [13] is a 15-item self-reported questionnaire developed to measure the burnout level of athletes. The ABQ includes three sub-scales, with five items per sub-scale: physical/emotional exhaustion, reduced sense of accomplishment, and sport devaluation. Each item is rated on a 5-point Likert scale type ranging from 1 (almost never) to 5 (almost always). A total score can be computed to measure the overall burnout of athletes. Higher scores indicate higher levels of burnout. Turkish validation of the scale demonstrated good evidence of reliability and validity [50]. In this study, Cronbach’s alpha coefficient for the ABQ was 0.85.

#### 2.2.3. Resilience

The Brief Resilience Scale (BRS) [51] is a 6-item self-report scale developed to assess the ability to bounce back or recover from setbacks, adversities, and failures. The items are answered on a 5-point Likert-type scale ranging from 1 (strongly disagree) to 5 (strongly agree). A total score can be computed by summing all items after reversing negatively worded items. Higher scores signify a higher ability of resilience to bounce back from stressful situations. Turkish adaptation of the scale revealed good evidence of reliability and validity [52]. In this study, Cronbach’s alpha coefficient for the BRS was 0.74.

#### 2.2.4. Motivation

The Sport Motivation Scale II (SMS-II; Pelletier et al., 2013) [40] was developed to measure people’s level of motivation regarding sports. The scale includes 18 items clustered into 6 dimensions (3 items per dimension): intrinsic, integrated, identified, introjected, external (or extrinsic), and amotivated. Only the extrinsic dimension was used for the purpose of this study. Each item was rated using a 7-point Likert scale ranging from 1 (does not correspond at all) to 7 (corresponds completely). Higher scores on the scale represent greater sport motivation. Turkish adaptation of the scale revealed good evidence of reliability and validity [53]. In this study, Cronbach’s alpha coefficient for the SMS-II was 0.82.

### 2.3. Procedure

A snowball sampling approach was used to collect the data. Athletes were requested to share the link with their friends after they took part in the study. The questionnaire was created in Google Forms and was made available to the participants through social networking sites (e.g., Twitter, Facebook, and WhatsApp) and e-mails from respective sports associations or clubs. All participants were informed about the nature, aims, and procedure of the study and they were asked to give their consent prior to completing the questionnaires. Participants were given information about their rights before and after participation through the first page of the online survey. In addition, they were fully assured of the confidentiality and anonymity of responses. Involvement in the study was completely voluntary and participants have not received any incentives for their involvement. The study procedure was in accordance with the Declaration of Helsinki [54] and institutional guidelines.

### 2.4. Statistical Analyses

In the proposed structural model, resilience and extrinsic motivation were considered as possible mediators (M) of the relationship between fear of failure (X) and athlete burnout (Y). We employed bootstrapping (10,000 samples) to examine the extent to which the fear of failure–athlete burnout relationship was mediated through resilience and extrinsic motivation [55,56]. To determine the indirect effect, a 95% bootstrapped confidence interval was computed [57]. Effect size (small = 0.01, medium = 0.09, and large = 0.25) was estimated to assess the emerging effect [58]. All analyses were performed using SPSS v26 for Windows and the macro-PROCESS v4.1 [55].

## 3. Results

### 3.1. Descriptive Statistics and Correlation Analysis

Table 2 presents descriptive statistics (i.e., mean and standard deviation), tests of normality (i.e., skewness and kurtosis), bivariate correlation coefficient, and internal consistency reliability estimates for the main variables of this study. The values of skewness (range = 0.12 and 0.53) and kurtosis (range = −0.59 and 0.45) indicated that all variables had a relatively normal distribution, with a commonly used reference for skewness and kurtosis values < |1| [59]. All the study variables had acceptable to good internal consistency reliability. The correlation analysis demonstrated that fear of failure had significant positive correlations with athlete burnout and extrinsic motivation, and a significant negative correlation with resilience. Athlete burnout was significantly negatively correlated with resilience and significantly positively correlated with extrinsic motivation. There was a significant negative relationship between resilience and extrinsic motivation.

### 3.2. Mediation Analysis

The results of the mediation analysis are presented in Table 3 and Table 4, and Figure 1. The results showed that fear of failure had a significant negative predictive effect on resilience (β = −0.31, *p* < 0.001) and a significant positive predictive effect on extrinsic motivation (β = 0.22, *p* < 0.001). Fear of failure explained 10% of the variance in resilience and 5% of the variance in extrinsic motivation. In addition, fear of failure (β = 0.19, *p* < 0.001), resilience (β = −0.37, *p* < 0.001), and extrinsic motivation (β = 0.11, *p* < 0.05) had significant predictive effects on athlete burnout. These three variables collectively explained 26% of the variance in athlete burnout. Furthermore, the indirect effect of fear of failure on athlete burnout was statistically significant through the mediating effects of resilience (effect = 0.18, 95% CI [0.10, 0.26]) and extrinsic motivation (effect = 0.04, 95% CI [0.00, 0.09]). These findings suggest that resilience and extrinsic motivation partially mediate the effect of fear of failure on athlete burnout.

## 4. Discussion

The purpose of this study was to explore the influence of fear of failure on athlete burnout and its potential mediating mechanisms of resilience and extrinsic motivation. As hypothesised, the results of this study indicated that fear of failure significantly negatively predicted resilience and significantly positively predicted extrinsic motivation and athlete burnout. This confirms the first research hypothesis. These results are consistent with the results of previous studies, showing the negative associations between the fear of failure and resilience [60] and positive associations between the fear of failure with motivation and burnout [61]. This may be because athletes with a high level of fear of failure are prone to avoid success and focus on failure when challenges arise [62]. Athletes with a low fear of failure are willing to spend time and energy, and maintain a positive attitude and interest in purposefully increasing effort towards a set goal, and this reduces extrinsic motivation and burnout [61].

The study also revealed that resilience negatively predicted the burnout of athletes, while extrinsic motivation positively predicted burnout, which validates the second hypothesis of this study. This result also supports the view that psychological resources and strengths (e.g., resilience and psychological capital) directly affect the results of individual development through the individual’s self-regulatory system [63,64,65]. The extrinsic motivation was found to increase burnout and diminish satisfaction [66]. Therefore, the promotion of resilience and the switching of the focus from extrinsic motivation to intrinsic motivation are important factors in contributing to reduced levels of burnout. Such promotion in sports helps to create a warm and supportive atmosphere, enabling athletes to feel more confident and supported to become more aware of their potential and own group members.

This study also found that resilience and extrinsic motivation play a parallel mediating role between the fear of failure and athlete burnout, which validates the third hypothesis. This result suggests that athletes with a high level of fear of failure have poor resilience and high extrinsic motivation, leading to a greater burnout experience. Earlier research has demonstrated that resilience is an important explanatory factor for the influence of negative feelings and experiences on mental health outcomes [67,68,69,70]. The cultivation of resilience can enhance athletes’ psychological health by reducing burnout experiences due to a fear of failure. Athletes with higher extrinsic motivation are more willing to actively participate in activities with the hope of achieving other people’s goals through rewards or external factors. This form of motivation is represented by a locus of external control, whose aim is either to fulfil an external demand or to acquire a reward [71], and is related to negative outcomes [44]. Therefore, the experience of fear of failure related to sports can diminish psychological resources, which in turn has the potential to deteriorate the psychological health of athletes, such as the greater experience of burnout.

From time to time, many people experience dysfunctional beliefs such as fear of failure, concern about the future, ruminating through past experiences, and self-blame about themselves. When dysfunctional beliefs or thinking are excessively experienced, they may lead to negative consequences [72,73]. While certain psychological resources (e.g., resilience) buffer the negative impacts of these beliefs on psychological health, others (e.g., extrinsic motivation) may exacerbate this impact. Researchers, practitioners, and policymakers could take the results of our study to help alleviate the suffering of individuals, including athletes, to help them grow and function effectively, not just at the individual level but also at the organisational level.

This study paves the way for numerous applications in activities, and not only those of athletes, but more generally including health promotion in the workplace. Burnout is a condition that can affect workers exposed to intense and prolonged stress which exceeds their individual resources and resilience strategies. Longitudinal studies conducted on frontline workers of COVID-19 hubs, forced to deal with the pandemic for a long time, have shown that the tendency to burnout increases with the constant increase in workload, compassion fatigue and isolation, and the lack of time for meditation, physical activity, and relaxation options [74]. During the COVID-19 pandemic, as well as in previous outbreaks, like the general public [75,76,77,78,79], frontline healthcare workers were at high risk of burnout, as are many healthcare workers outside of epidemics who lack adequate resilience and ongoing motivation [80,81].

Although the current study presents new information relative to the mediating roles of resilience and extrinsic motivation on the relationship between fear of failure and burnout, it is not without limitations. The cross-sectional research design of this research did not enable firm causal conclusions among the variables. Carrying out longitudinal research by collecting data at different time points alongside experimental research is necessary for future studies, as there are various dynamic and complex processes by which fear of failure is associated with psychological outcomes. Additionally, the most salient mediating processes appear to involve psychological strengths such as resilience, which need to be examined further in subsequent studies. In addition, the data for this study were collected through an online survey. In spite of their superiority, such as for reasons of accessibility and affordability, online surveys have been criticised in terms of carrying selection bias and challenges to including participants who do not have access to the internet [82]. Finally, there was a substantial difference between the proportion of male (93.43%) and female athletes (6.57%). The results of the current sample might not be representative of the population. Therefore, future research needs to conduct studies with an ideally equal number of male and female athletes to improve the representativeness of the sample. 

## 5. Conclusions

In conclusion, the present study revealed that fear of failure may have affected symptoms of athlete burnout. However, this can be explained via the impacts of resilience and extrinsic motivation. While extrinsic motivation serves as a risk factor in terms of its impact on athlete burnout, resilience plays an important role in protecting athletes’ psychological health by reducing the adverse impact of fear of failure on athlete burnout. The examination of protective factors alongside risk factors can provide a wider insight into measures to cope with the symptoms of burnout in athletes resulting from fear of failure related to sports. The findings of the present study are also useful for sports coaching to help in developing health promotion programs and cultivating resilience among the athlete population. Additionally, it will be the responsibility of policymakers to develop public health strategies through the promotion of policies related to psychological strengths in the context of sports.

## Figures and Tables

**Table 1:** Descriptive characteristics of the participants.

Variable	Level	*n*	%
Gender	Males	313	93.43
	Females	22	6.57
Marital status	Single	242	72.24
	Married	92	27.46
	Divorced/Widowed	1	0.30
The highest level of education	High school or below	140	41.79
	Bachelor’s degree	176	52.54
	Postgraduate	19	5.67
Active sports years	1–3 years	42	12.54
	4–6 years	82	24.48
	7–9 years	55	16.42
	10 years and above	156	46.57
Best sport rating	District Championship	94	28.06
	National Championship	124	37.01
	European Championship	51	15.22
	World Championship	38	11.34
	None	22	6.57
	Unanswered	6	1.79
National level of athletes	National	168	50.15
	Non-national	167	49.85

**Table 2:** Descriptive statistics and correlations.

	Descriptive Statistics					Correlation			
Variable	*M*	*SD*	Skew	Kurt	α	1	2	3	4
1. Fear of failure	21.12	5.66	0.12	−0.09	0.82	—	0.33 **	−0.31 **	0.22 **
2. Athlete burnout	30.04	8.61	0.53	0.35	0.85		—	−0.45 **	0.23 **
3. Resilience	19.65	3.71	0.48	0.45	0.74			—	−0.19 **
4. Extrinsic motivation	9.99	4.58	0.30	−0.59	0.82				—

Note. ** *p* < 0.01.

**Table 3:** Unstandardised coefficients for the mediation model.

		Consequence			
		*M*_1_ (Resilience)			
Antecedent		Coeff.	*SE*	*t*	*p*
*X* (Fear of failure)	*a*_1_	–0.20	0.03	–5.96	<0.001
Constant	*i*_M1_	23.95	0.75	32.10	<0.001
		*R*^2^ = 0.10 *F* = 35.57; *p* < 0.001			
		*M*_2_ (Extrinsic motivation)			
*X* (Fear of failure)	*a*_2_	0.18	0.04	4.17	<0.001
Constant	*i*_M2_	6.18	0.94	6.55	<0.001
		*R*^2^ = 0.05 *F* = 17.40; *p* < 0.001			
		*Y* (Athlete burnout)			
*X* (Fear of failure)	*c*’	0.29	0.08	3.73	<0.001
*M*_1_ (Resilience)	*b*_1_	–0.87	0.12	–7.44	<0.001
*M*_2_ (Extrinsic motivation)	*b*_2_	0.21	0.09	2.30	<0.05
Constant	*i*_y_	38.92	3.32	11.72	<0.001
		*R*^2^ = 0.26 *F* = 38.36; *p* < 0.001			

Note. *SE* = standard error. Coeff = unstandardised coefficient. *X* = independent variable; *M* = mediator variable; *Y* = dependent variable.

**Table 4:** Standardised indirect effects.

Path	Effect	*SE*	BootLLCI	BootULCI
Total indirect effect	0.21	0.05	0.13	0.32
Fear of failure –> Resilience –> Athlete burnout	0.18	0.04	0.10	0.26
Fear of failure –> Extrinsic motivation –> Athlete burnout	0.04	0.02	0.00	0.09

Note. The number of bootstrap samples for percentile bootstrap confidence intervals: 10,000.

## Data Availability

The data that support the findings of this study are available from the corresponding author (M.Y.) upon reasonable request.
